# Indole alkaloids inhibit zika and chikungunya virus infection in different cell lines

**DOI:** 10.1186/s12906-021-03386-z

**Published:** 2021-08-28

**Authors:** Laura Milena Monsalve-Escudero, Vanessa Loaiza-Cano, Yina Pájaro-González, Andrés Felipe Oliveros-Díaz, Fredyc Diaz-Castillo, Wiston Quiñones, Sara Robledo, Marlen Martinez-Gutierrez

**Affiliations:** 1 Grupo de Investigación en Ciencias Animales-GRICA. Facultad de Medicina Veterinaria y Zootecnia, Universidad Cooperativa de Colombia, Bucaramanga, Colombia; 2 Laboratorio de Investigaciones Fitoquímicas y Farmacológicas de la Universidad de Cartagena – LIFFUC, Universidad de Cartagena, Cartagena, Colombia; 3 Grupo de Investigación en Farmacia Asistencial y Farmacología, Universidad del Atlántico, Barranquilla, Colombia; 4 Grupo de Química Orgánica de Productos Naturales. Universidad de Antioquia, Medellín, Colombia; 5 Programa de Estudio y Control de Enfermedades Tropicales-PECET, Universidad de Antioquia, Medellín, Colombia

**Keywords:** Zika virus, Chikungunya virus, Indole alkaloids, Molecular docking, Antiviral\

## Abstract

**Background:**

In recent years, an increase in the occurrence of illnesses caused by two clinically- important arboviruses has been reported: Zika virus (ZIKV) and Chikungunya virus (CHIKV). There is no licensed antiviral treatment for either of the two abovementioned viruses. Bearing in mind that the antiviral effect of indole alkaloids has been reported for other arboviral models, the present study proposed to evaluate the antiviral in vitro and in silico effects of four indole alkaloids on infections by these two viruses in different cell lines.

**Methods:**

The antiviral effects of voacangine (VOAC), voacangine-7-hydroxyindolenine (VOAC-OH), rupicoline and 3-oxo voacangine (OXO-VOAC) were evaluated in Vero, U937 and A549 cells using different experimental strategies (Pre, Trans, Post and combined treatment). Viral infection was quantified by different methodologies, including infectious viral particles by plating, viral genome by RT-qPCR, and viral protein by cell ELISA. Moreover, molecular docking was used to evaluate the possible interactions between structural and nonstructural viral proteins and the compounds. The results obtained from the antiviral strategies for each experimental condition were compared in all cases with the untreated controls. Statistically significant differences were identified using a parametric Student’s *t*-test. In all cases, *p* values below 0.05 (*p* < 0.05) were considered statistically significant.

**Results:**

In the pre-treatment strategy in Vero cells, VOAC and VOAC-OH inhibited both viral models and OXO-VOAC inhibited only ZIKV; in U937 cells infected with CHIKV/Col, only VOAC-OH inhibited infection, but none of the compounds had activity in A549 cells; in U937 cells and A549 cells infected with ZIKV/Col, the three compounds that were effective in Vero cells also had antiviral activity. In the trans-treatment strategy, only VOAC-OH was virucidal against ZIKV/Col. In the post-treatment strategy, only rupicoline was effective in the CHIKV/Col model in Vero and A549 cells, whereas VOAC and VOAC-OH inhibited ZIKV infection in all three cell lines. In the combined strategy, VOAC, VOAC-OH and rupicoline inhibited CHIKV/Col and ZIKV/Col, but only rupicoline improved the antiviral effect of ZIKV/Col-infected cultures with respect to the individual strategies. Molecular docking showed that all the compounds had favorable binding energies with the structural proteins E2 and NSP2 (CHIKV) and E and NS5 (ZIKV).

**Conclusions:**

The present study demonstrates that indole alkaloids are promising antiviral drugs in the process of ZIKV and CHIKV infection; however, the mechanisms of action evaluated in this study would indicate that the effect is different in each viral model and, in turn, dependent on the cell line.

**Supplementary Information:**

The online version contains supplementary material available at 10.1186/s12906-021-03386-z.

## Background

Arboviruses are viruses that require the presence of a female hematophagous arthropod to be transmitted between vertebrate hosts [1]. In recent years, an increase has been reported in the occurrence of illnesses caused by two clinically-important arboviruses: Chikungunya fever caused by Chikungunya virus (CHIKV) and Zika fever caused by Zika virus (ZIKV). The transmission of these arboviruses occurs by the same vectors, and their simultaneous circulation occurs in several regions of the world, with previously reported cases of co-infections [2], some of which have been associated with mortality in some cases [3].

First, CHIKV belongs to the genus *Alphavirus* and *Togaviridae* family. CHIKV is an enveloped icosahedral virus with a diameter of 60–70 nm, with a positive-sense single-stranded RNA genome of approximately 12 kb in length with two open reading frames (ORFs). The first ORF gives rise to four nonstructural proteins (NSP1, NSP2, NSP3, and NSP4) that are translated from genomic RNA, and the second ORF encodes five structural proteins (capsid protein C and glycoproteins E1, E2, E3, and 6 K) that are translated from subgenomic RNA [4]. This virus has been the cause of major epidemics in recent decades. One of the most significant epidemics occurred in late 2014 in the Pacific Islands, and it caused more than one million cases in less than 4 months in the Caribbean Islands, Latin American countries, and the United States in the same year [5]. Although CHIKV infection is an acute debilitating self-limiting sickness characterized by intense polyarthralgia, it may leave chronic long-term articular and rheumatic manifestations with a significant loss of quality of life in some cases [6]. Over time, unexpected delayed rheumatic complications can manifest years after acute CHIKV infection, particularly with the development of potentially destructive rheumatism [7] .

On the other hand, ZIKV is an icosahedral virus that belongs to the *Flaviviridae* family. It has a lipid envelope and a positive-sense single-stranded RNA genome composed of a single ORF that encodes a single polyprotein [8]. This one is cleaved into three structural capsid proteins (C), the membrane precursor (prM), the envelope protein (E), and seven nonstructural proteins (NS1, NS2a, NS2b, NS3, NS4a, NS4b, and NS5) [9]. In 2016, the WHO declared ZIKV infection an international public health emergency because of the rise in the number of cases [10]. As in other arboviruses, the illness produced by this virus is characterized by a mild fever, but it can lead to the development of serious sequelae such as microcephaly, brain calcifications [11], and Guillain–Barré syndrome [12].

As described above, the importance of infection-transmitting vectors (*A. aegypti* and *A. albopictus*) in the tropical and subtropical areas of the world has led to the main strategy of control and prevention of these illnesses, community-based interventions to prevent their reproduction [13]. However, the emergence of resistance to insecticides and the lack of coverage of the programs implemented by government agencies for the control and eradication of the vector lead to control that is not entirely effective [14]. The second control strategy should be vaccination, but although progress has been made, with the development and clinical evaluation of several candidates, none of them has passed Phase III; therefore, no vaccine has been approved [15–17]. Finally, the third strategy to control the infection of these viruses is the search for compounds with antiviral activity [18]. Despite a growing number of studies in this area, there are still no licensed drugs available [17, 19].

Although several antiviral approaches have been developed in recent decades, the evaluation of compounds obtained from natural sources is promising as the basis for the production of new treatments against some viruses [20], including alkaloids. One of the most alkaloid-rich plant families is the family *Apocynaceae* [21], which includes the species *Tabernaemontana cymosa* (*T. cymosa*) [22]. Indole alkaloids (which possess a pyrrole adjacent to the benzene ring) are compounds that originate from the tryptophan-derived secondary metabolites of some plants and have shown antiviral potential, such as the drug arbidol (umifenovir), which is effective against influenza A [23], TMC647055, a compound that inhibits the hepatitis C virus [24] and INDOPY-1, an inhibitor of human immunodeficiency virus type 1 [25]. Additionally, our research team has shown that voacangine derived from the plant *T. cymosa* inhibits the replication of the viral genome of DENV-2 [26] and that some of its structural analogs (VOAC-OH and rupicoline) have antiviral and virucidal effects depending on the serotype and/or DENV strain [27] .

Considering the antecedents, the prevalence in ZIKV and CHIKV cases worldwide, the lack of therapeutic alternatives for these viral infections, and the use of alkaloids as a source of antivirals, this study proposed to evaluate the antiviral in vitro and in silico effects of four indole alkaloids against CHIKV and ZIKV in different cell lines.

## Methods

### Compounds

In this study, no plant parts were used. All evaluations were performed with four compounds from the seeds of *T. cymosa* that had been previously isolated and reported: VOAC [26], VOAC-OH, rupicoline and OXO-VOAC [27]. These compounds are subject to contracts for access to genetic resources and derived products #130 of 2016 (RGE0176) and #292 of 2020 (RGE0343) signed with the Ministry of Environment and Sustainable Development of the Republic of Colombia. Suramin, ribavirin, or heparin were used as positive inhibition controls depending on the antiviral strategy, the type of virus and the cell line.

### Cell and virus maintenance

C6/36 cells were donated by Dr. Raquel Ocazionez (Universidad Industrial de Santander. Bucaramanga, Colombia) and kept at 28 °C and cultured in Leibovitz medium (L-15) supplemented with 10% fetal bovine serum (FBS, GIBCO®) and 20 mM HEPES. The Vero cell line was donated by Dr. Jorge Osorio, Department of Pathobiological Sciences, University of Wisconsin (Madison, WI, United States) and used as a screening model for the compounds; the U937 and A549 cell lines were donated by Dr. Jaime Castellanos (Universidad El Bosque. Bogotá, Colombia) and Dr. Carolina Quintero (Universidad Nacional de Colombia. Medellin, Colombia), respectively, were used as models for some antiviral assays. These cell lines were cultured in Dulbecco’s modified Eagle’s medium (GIBCO®) supplemented with 2% FBS and a 1% antibiotic/antimycotic solution (10 mg/ml streptomycin, 10,000 U/ml penicillin, and 0.025 mg/ml amphotericin B, GIBCO®) and kept in a humid environment with 5% CO2 at 37 °C. The maintenance of the cultures corresponded to standard protocols recommended by the cell supplier. All experiments were performed at a multiplicity of infection of one (1) with two Colombian clinical isolates. CHIKV/Col [26] and ZIKV/Col [28] previously reported.

### Determination of the viability of indole alkaloids

The 3-[4,5-dimethylthiazol-2-yl]-2,5 diphenyl tetrazolium bromide (MTT) assay [29] was used to evaluate the viability of the indole alkaloids and inhibition controls at a single concentration for each compound on Vero, U937 and A549 cells seeded in 96-well plates for 48 h. These concentrations were defined based on previous reports obtained by our investigation group [27]. Absorbance reading was performed on a Multiskan™ FC Microplate Photometer (Thermo Scientific) at 450 nm. In all cases, compound-free cultures were used to represent 100% viability. Each experiment was performed by two independent experimental units with three replicas each (n: 4).

### In vitro evaluations

The evaluation of in vitro antiviral activity was performed using four previously described strategies: Pre, Trans, Post and combined treatment [27]. In all cases, 6.0 × 10^4^ cells were seeded per well, a single noncytotoxic concentration of the compounds was used, and the monolayers were inoculated for 2 h with each virus evaluated in all the treatment strategies. Viral replication was allowed for 48 h for the individual strategies and 24 h for the combined strategy. The supernatants and monolayers were collected and stored at − 80 °C until processing. The compounds that demonstrated antiviral activity in the pre-treatment or post-treatment strategies were evaluated using the same strategies in the U937 and A549 cell lines. For the compounds that showed post-treatment activity, the number of genomic copies/mL and viral protein were quantified by qPCR and in-cell enzyme-linked immunosorbent assay (Cell ELISA), respectively.

### Quantification of infectious viral particles by plaque assay

Serial dilutions of the supernatants were inoculated for 2 h on monolayers of Vero cells (1.2 × 10^4^/well). Afterwards, the viral inoculum was removed, and 1.5% carboxymethylcellulose (Sigma-Aldrich) was added. The monolayers were fixed with 4% paraformaldehyde (Sigma-Aldrich) and stained with crystal violet after 4 or 7 days of incubation depending on the virus (CHIKV/Col or ZIKV/Col, respectively).

### Quantification of intracellular viral genome by qPCR

RNA was extracted from the infected and treated monolayers following the manufacturer’s instructions (Zymo Quick-RNA Viral Kit). Reverse transcription was performed from 500 ng of RNA using the M-MLV Reverse Transcriptase kit. qPCR was performed with the POWERUP™ SYBR™ Green Master Mix kit (Thermo Fisher Scientific) in QuantStudio 3 using previously described primers and protocols [28]. For the absolute quantification of the genomic copies, we used standard curves of plasmids previously constructed by our group.

### Intracellular quantification of viral protein

The monolayers were fixed with PFA 4% and then permeabilized with Triton X-100 (0.1%) for 30 min and then treated with 0.3% H2O2 prepared in 10% methanol for 30 min; nonspecific sites were then blocked with FBS (10%) for 30 min. Subsequently, the primary anti-ZIKV and anti-CHIKV antibodies previously reported [28] were incubated overnight at 4 °C. The secondary antibody coupled to horseradish peroxidase was then added for 30 min, and TMB was finally added to read the absorbance at 620 nm (Multiskan™ FC Microplate Photometer, Thermo Scientific).

### In silico evaluations

The three-dimensional structures of one structural protein and one nonstructural protein for each viral model were obtained from the Protein Data Bank (PDB) database with a resolution of less than 3 Å. Envelope domain III (PDB: 5JHM) and the NS5 polymerase domain (PDB: 5 U04) were used for ZIKV; the E2 protein (PDB: 3 N44) and NSP2 protease (PDB: 3TRK) were used for CHIKV and treated with Python Molecular Viewer (PMV) [30] as previously reported [31]. The site of interaction of the compounds with the proteins was identified using the PeptiMap tool [32, 33]. The compounds were obtained and treated as previously reported [27]. The binding free energies of the compound–protein interactions were obtained by AutoDock Vina software [34]. The intermolecular interactions (hydrogen bonds and hydrophobic interactions) were identified from two-dimensional diagrams using LigPlot® software v1.4.5 (https://www.ebi.ac.uk/thornton-srv/software/LIGPLOT/) [35]. Finally, molecular dynamics was performed using GROMACS software to evaluate the stability over time (50 ns) of the complex formed with virucidal compounds and the favorable energy of binding in AutoDock Vina for the E protein [27]. The results obtained include the evaluation of the resulting trajectories and a plot of distances during the evaluated time.

### Statistical analysis

The normality of the data was evaluated using the Shapiro–Wilk test. The results obtained from the antiviral strategies for each experimental condition were compared in all cases with the infected untreated controls. Statistically significant differences were identified using a parametric Student’s *t*-test (data with normal distribution). All statistical analyses were performed using the Prism® 7.01 package for Windows™ (GraphPad Software, San Diego, CA). In all cases, *p* values below 0.05 (*p* < 0.05) were considered statistically significant.

## Results

### The viability of different cell lines was not affected by indole alkaloids

The viability of indole alkaloids and inhibition controls was evaluated in Vero, U937 and A549 cells by the MTT method. Rupicoline was the compound with the best viability percentages in all three cell lines, 94.5, 99.4 and 99.3%, for Vero, U937 and A549 cells, respectively. The lowest viability percentage in Vero cells was obtained with VOAC-OH, 75.9%; in U937 cell line with OXO-VOAC, 77.0%; and in A549, VOAC with 84.7%. Nevertheless, in all cases, the viabilities were adequate for subsequent antiviral work. None of the inhibition controls had viabilities below 75%. (Table 1).

**Table 1:** Viability percentage of indole alkaloids in Vero, U937 and A549 cell lines

		***Viability Percentages***		
Compound	Concentration	Vero cells	U937 cells	A549 cells
*Suramin*	125.0 μM	93,5 ± 7,6	93.6 ± 0.9	98.3 ± 2.7
*Heparin*	17.6 μM	94,3 ± 2,3	95.2 ± 1.6	92.8 ± 0.9
*Ribavirin*	200.0 μM	92,2 ± 2,9	91.8 ± 1.2	84.5 ± 2.2
*VOAC*	17.1 μM	86.1 ± 1.9	88.8 ± 0.5	84.7 ± 1.7
*VOAC-OH*	16.4 μM	75.9 ± 3.8	91.3 ± 1.5	95.0 ± 2.7
*Rupicoline*	16.4 μM	94.5 ± 3.7	99.4 ± 1.3	99.3 ± 2.8
*OXO-VOAC*	16.5 μM	90.6 ± 1.3	77.0 ± 1.2	89.8 ± 2.0

### The antiviral effect of VOAC and VOAC-OH in the pre-treatment strategy was dependent on the cell line in the CHIKV/col infection model

**Fig. 1:**
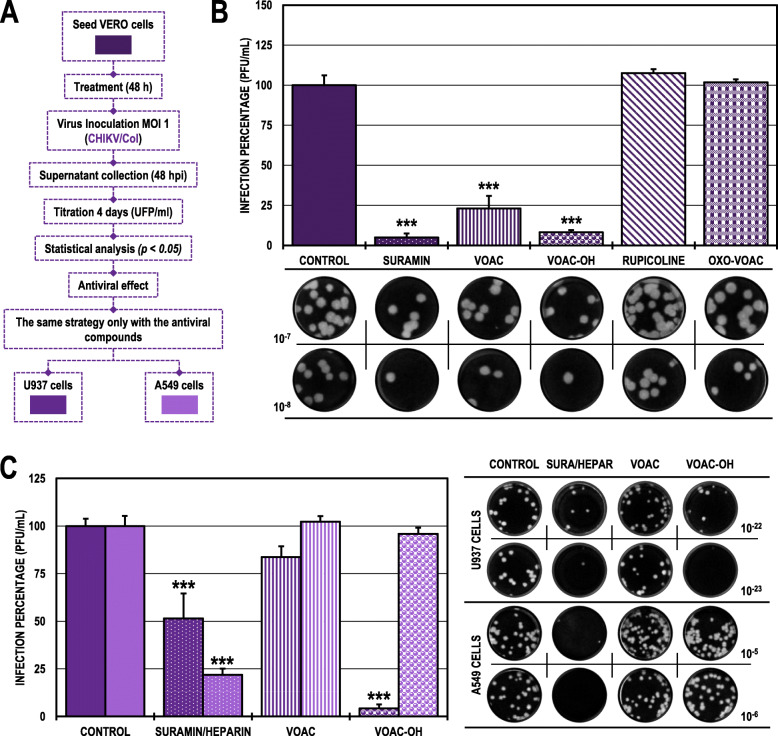
Antiviral effects on the production of infectious viral particles from cultures treated and posteriorly infected with CHIKV/Col. **A.** Schematic representation of the pre-treatment strategy explained in the Materials and Methods section. **B.** Infection percentage calculated according to the results obtained by plaque assay (PFU/ml) of the supernatants collected from Vero cells under each experimental condition. **C.** Infection percentage calculated according to the results obtained by plaque assay (PFU/ml) of the supernatants collected from U937 cells (dark purple bars) and A549 cells (light purple bars). The asterisks indicate statistically significant differences with respect to the untreated control (*p < 0.05, **p < 0.01 and ***p < 0.001; Student’s *t-*test), and error bars indicate the standard error of the mean; n: 4. Representative images of plaques formed under each experimental condition are shown (**B** and **C**)

In Vero cell cultures treated and subsequently infected with CHIKV/Col (pre-treatment strategy to detect possible activity in the early stages of infection or host-directed activity through modulation of cellular processes) (Fig. 1A), only the alkaloids VOAC and VOAC-OH significantly decreased the number of infectious particles compared with the untreated control, with infection rates of 23.1 and 8.2%, respectively (Fig. 1B). The evaluation of the two most promising compounds for CHIVK/Col with this strategy (VOAC and VOAC-OH) in the U937 and A549 cell lines showed different results. Only VOAC-OH significantly inhibited infection to 4.2% in the U937 cells compared with the untreated control, and none of the compounds had an antiviral effect in the A549 cell line. Suramin was the positive inhibitory control in the Vero and U937 lines (5.0 and 51.5%, respectively), and heparin was the positive inhibitory control in the A549 line (21.9%) (Fig. 1C; Table 2).

**Table 2:** Viral titers (PFU/ml) obtained in all treatment strategies with indole alkaloids

Treatment Strategy	Virus	Cell Line	Untreated control	Inhibition control		VOAC	VOAC-OH	RUPICOLINE	OXO-VOAC
*Pre-treatment* *(UFP/ml)*	*CHIKV/Col*	Vero	2,65 × 10^8^	Suramin	1,31 × 10^7^	6,13 × 10^7^	2,18 × 10^7^	2,85 × 10^8^	2,70 × 10^8^
		U937	7,40 × 10^24^	Suramin	3,81 × 10^24^	6,19 × 10^24^	3,13 × 10^23^	N/A	N/A
		A549	1,98 × 10^6^	Heparin	4,33 × 10^5^	2,02 × 10^6^	1,90 × 10^6^	N/A	N/A
	*ZIKV/Col*	Vero	4,95 × 10^3^	Suramin	4,65 × 10^2^	6,85 × 10^2^	5,43 × 10^2^	5,90 × 10^3^	2,81 × 10^3^
		U937	7,35 × 10^4^	Suramin	2,00 × 10^4^	< 1,00 × 10^0^	3,75 × 10^3^	N/A	6,06 × 10^4^
		A549	7,00 × 10^4^	Suramin	1,31 × 10^4^	1,06 × 10^4^	1,25 × 10^3^	N/A	5,44 × 10^4^
*Trans-treatment* *(UFP/ml)*	*CHIKV/Col*	Vero	1,94 × 10^8^	Suramin	4,93 × 10^7^	1,60 × 10^8^	2,18 × 10^8^	2,10 × 10^8^	1,52 × 10^8^
	*ZIKV/Col*	Vero	5,17 × 10^4^	Suramin	2,20 × 10^3^	5,95 × 10^4^	3,93 × 10^3^	5,23 × 10^4^	5,58 × 10^4^
*Post-treatment* *(UFP/ml)*	*CHIKV/Col*	Vero	4,33 × 10^8^	Ribavirin	2,43 × 10^6^	4,10 × 10^8^	4,25 × 10^8^	3,90 × 10^7^	4,12 × 10^8^
		U937	1,94 × 10^12^	Ribavirin	9,23 × 10^11^	N/A	N/A	1,73 × 10^12^	N/A
		A549	7,96 × 10^11^	Ribavirin	3,43 × 10^11^	N/A	N/A	5,03 × 10^11^	N/A
	*ZIKV/Col*	Vero	2,30 × 10^5^	Ribavirin	< 1,00 × 10^0^	4,43 × 10^4^	2,68 × 10^4^	1,90 × 10^5^	1,92 × 10^5^
		U937	1,01 × 10^7^	Ribavirin	2,75 × 10^6^	< 1,00 × 10^0^	< 1,00 × 10^0^	N/A	N/A
		A549	8,30 × 10^6^	Ribavirin	3,19 × 10^6^	< 1,00 × 10^0^	< 1,00 × 10^0^	N/A	N/A
*Post-treatment* *(Genome copies/ml)*	*CHIKV/Col*	Vero	1,12 × 10^5^	Ribavirin	2,96 × 10^4^	N/A	N/A	5,73 × 10^5^	N/A
		U937	N/A	N/A	N/A	N/A	N/A	N/A	N/A
		A549	1,15 × 10^3^	Ribavirin	6,12 × 10^2^	N/A	N/A	1,10 × 10^3^	N/A
	*ZIKV/Col*	Vero	1,17 × 10^7^	Ribavirin	6,05 × 10^5^	2,04 × 10^7^	1,18 × 10^7^	N/A	N/A
		U937	4,76 × 10^6^	Ribavirin	2,05 × 10^6^	1,46 × 10^5^	1,43 × 10^5^	N/A	N/A
		A549	1,41 × 10^7^	Ribavirin	2,22 × 10^6^	2,30 × 10^5^	2,28 × 10^5^	N/A	N/A
*Combined* *(UFP/ml)*	*CHIKV/Col*	Vero	1,97 × 10^6^	Suramin	< 1,00 × 10^0^	4,00 × 10^5^	1,50 × 10^5^	1,88 × 10^5^	1,93 × 10^6^
	*ZIKV/Col*	Vero	4,22 × 10^4^	Suramin	< 1,00 × 10^0^	4,44 × 10^3^	< 1,00 × 10^0^	2,50 × 10^3^	3,09 × 10^4^

### The antiviral effect of VOAC and VOAC-OH in the pre-treatment strategy was independent of the cell line in the ZIKV/col infection model

**Fig. 2:**
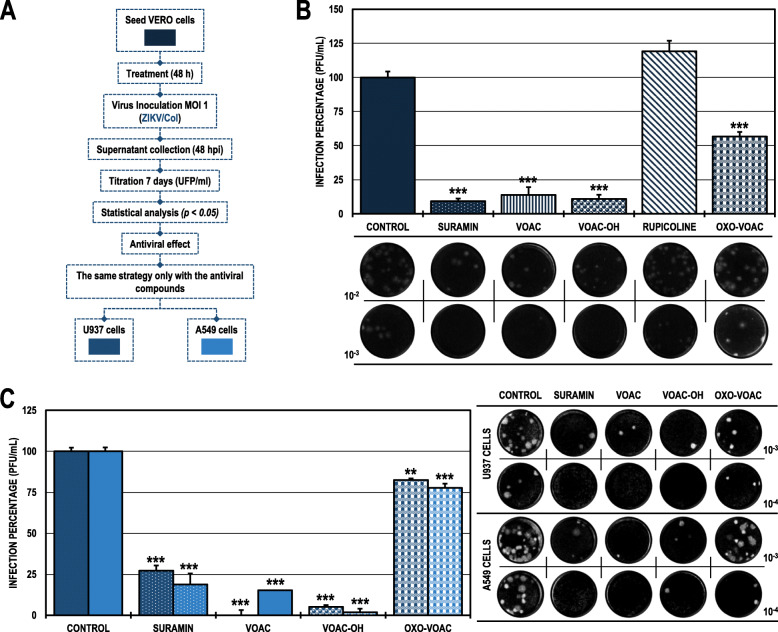
Antiviral effects on the production of infectious viral particles from cultures treated and posteriorly infected with ZIKV/Col. **A.** Schematic representation of the pre-treatment strategy explained in the Materials and Methods section. **B.** Infection percentage calculated according to the results obtained by plaque assay (PFU/ml) of the supernatants collected from Vero cells under each experimental condition. **C.** Infection percentage calculated according to the results obtained by plaque assay (PFU/ml) of the supernatants collected from U937 cells (dark blue bars) and A549 cells (light blue bars). The asterisks indicate statistically significant differences with respect to the untreated control (*p < 0.05, **p < 0.01 and ***p < 0.001; Student’s *t*-test), and error bars indicate the standard error of the mean; n: 4. Representative images of plaques formed under each experimental condition are shown (**B** and **C**)

When we evaluated the same strategy (pre-treatment) in the ZIKV/Col model (Fig. 2A), we found a statistically significant decrease in the cultures treated with the same compounds that inhibited CHIKV/Col, VOAC and VOAC-OH and in those treated with OXO-VOAC compared with the untreated control. In those cases, the infection rates were 13.8, 11.0, and 56.7%, respectively (Fig. 2B). In both cases, suramin was used as a positive inhibition control, decreasing the infection percentage to 9.4% for ZIKV/Col. On the other hand, the evaluation of the three compounds that had an antiviral effect in the ZIKV/Col infection model on Vero cells (VOAC, VOAC-OH, and OXO-VOAC) maintained a significant antiviral effect on both cell lines: U937 (infection rates of 0.0, 5.1 and 82.5%) and A549 (infection rates of 15.2, 1.8 and 77.7%), respectively. Suramin was the positive inhibitory control in the U937 cell line, and heparin was the positive inhibitory control in the A549 cell line (27.2 and 7.1%, respectively) (Fig. 2C; Table 2).

### Only VOAC-OH was virucidal against ZIKV/col

**Fig. 3:**
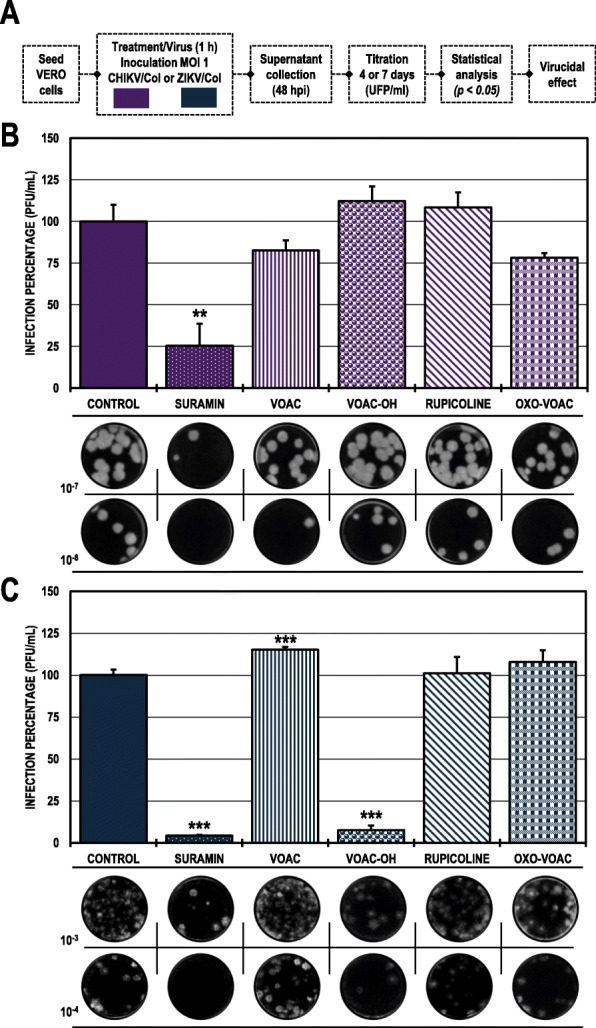
Virucidal effect against CHIKV/Col and ZIKV/Col. **A.** Schematic representation of the trans-treatment strategy explained in the Materials and Methods section. **B.** Infection percentage calculated according to the results obtained by plaque assay (PFU/ml) of the supernatants collected from Vero cells infected with CHIKV/Col under each experimental condition. **C.** Percentage calculated according to the results obtained by plaque assay (PFU/ml) of the supernatants collected from Vero cells infected with ZIKV/Col under each experimental condition. The asterisks indicate statistically significant differences with respect to the control without compound (*p < 0.05, **p < 0.01 and ***p < 0.001; Student’s *t*-test), and error bars indicate the standard error of the mean; n: 4. Representative images of plaques formed under each experimental condition are shown (**B** and **C**)

When the compounds were put in direct contact with the virus (trans-treatment, Fig. 3A) to evaluate their possible virucidal activity, CHIKV/Col did not inhibit the production of infectious viral particles compared with the untreated control (Fig. 3B). Meanwhile, in the cultures inoculated with the mixture of ZIKV/Col and VOAC-OH, the number of infectious particles significantly decreased to a 7.6% infection rate compared with the untreated control. Suramin was used as a positive inhibition control for both infection models (25.5 and 4.3%, respectively) (Fig. 3C; Table 2).

### The antiviral effect of rupicoline in the post-treatment strategy was dependent on the cell line in the CHIKV/col infection model

**Fig. 4:**
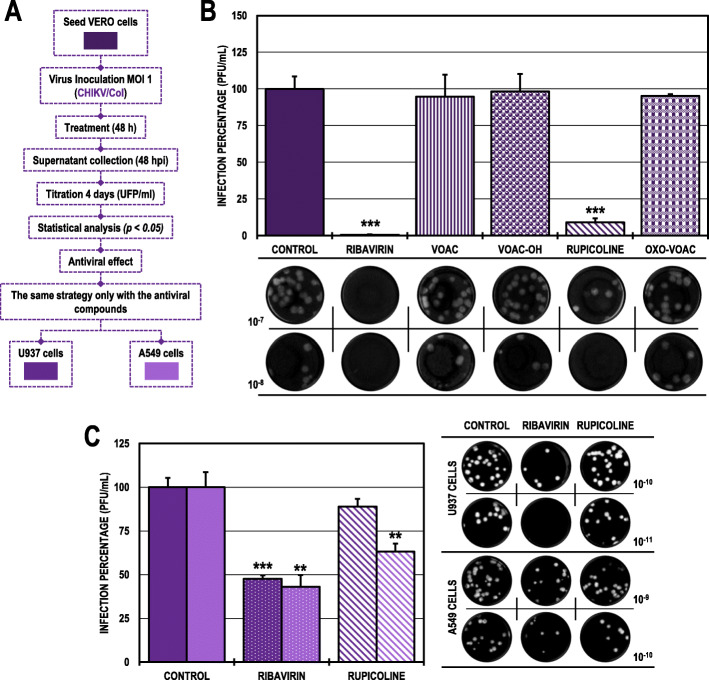
Antiviral effects on the production of infectious viral particles from cultures infected with CHIKV/Col and posteriorly treated. **A.** Schematic representation of the post-treatment strategy explained in the Materials and Methods section. **B.** Infection percentage calculated according to the results obtained by plaque assay (PFU/ml) of the supernatants collected from Vero cells under each experimental condition. **C.** Percentage calculated according to the results obtained by plaque assay (PFU/ml) of the supernatants collected from U937 cells (dark purple bars) and A549 cells (light purple bars). The asterisks indicate statistically significant differences with respect to the control without compound (*p < 0.05, **p < 0.01 and ***p < 0.001; Student’s *t*-test), and error bars indicate the standard error of the mean; n: 4. Representative images of plaques formed under each experimental condition are shown (**B** and **C**)

In the Vero cell cultures infected with CHIKV/Col and subsequently treated (Fig. 4A), only rupicoline significantly inhibited the infection to 9.0% compared with the untreated control (Fig. 4B). In contrast, this same compound only inhibited CHIKV infection in the A549 cell line to 63.1% but not in the U937 cell line (Fig. 4C). Ribavirin was the positive inhibitory control in all cell lines (0.6, 47.6 and 43.0%, respectively). (Table 2).

### The antiviral effect of VOAC and VOAC-OH in the post-treatment strategy was independent of the cell line in the ZIKV/col infection model

**Fig. 5:**
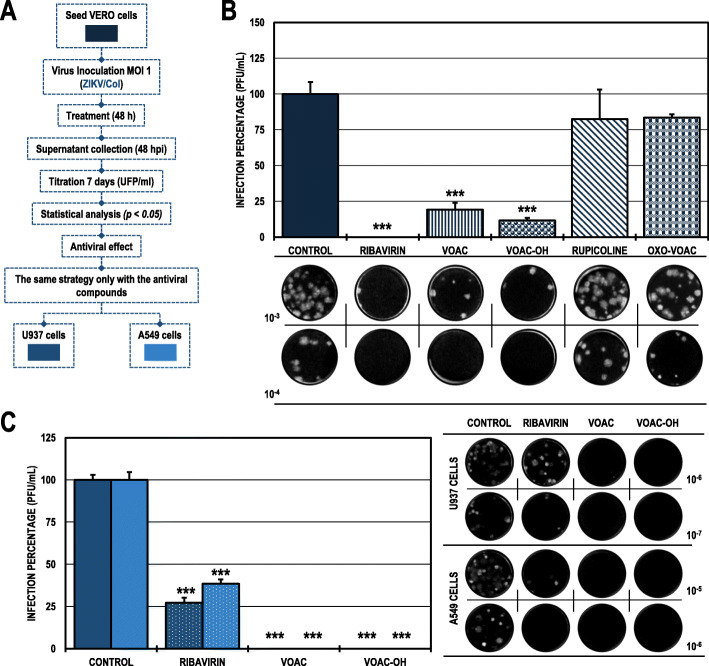
Antiviral effects on the production of infectious viral particles from cultures infected with ZIKV/Col and posteriorly treated. **A.** Schematic representation of the post-treatment strategy explained in the Materials and Methods section. **B.** Infection percentage calculated according to the results obtained by plaque assay (PFU/ml) of the supernatants collected from Vero cells under each experimental condition. **C.** Percentage calculated according to the results obtained by plaque assay (PFU/ml) of the supernatants collected from U937 cells (dark blue bars) and A549 cells (light blue bars). The asterisks indicate statistically significant differences with respect to the control without compound (*p < 0.05, **p < 0.01 and ***p < 0.001; Student’s *t*-test), and error bars indicate the standard error of the mean; n: 4. Representative images of plaques formed under each experimental condition are shown (**B** and **C**)

In the post-treated ZIKV/Col-infected Vero cells (Fig. 5A), the inhibitory compounds were VOAC and VOAC-OH with infection percentages of 19.2 and 11.6%, respectively, compared with the untreated control (Fig. 5B). These same compounds also inhibited ZIKV infection in both U937 and A549 cell lines to an infection rate of 0.0% in all cases (Fig. 5C). Ribavirin was used as an inhibitory control in all three cell lines (0.0% in Vero cells; 27.2% in U937 cells and 38.4% in A549 cells) (Table 2).

### The effect of the compounds on genome replication and viral protein production was cell-dependent according to the viral model

**Fig. 6:**
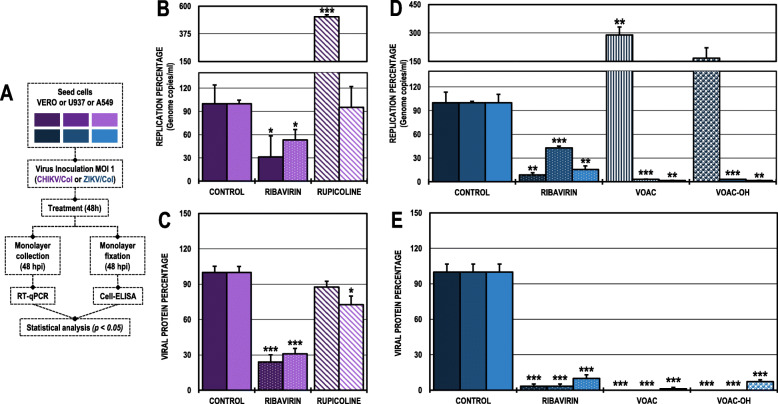
Effect on intracellular genomic copies and viral protein using the post-treatment strategy in different cell lines. **A.** Schematic representation of the post-treatment strategy explained in the Materials and Methods section. **B.** The replication percentage was calculated according to the results obtained by RT-qPCR of monolayers infected with CHIKV/Col. **C.** Viral protein percentage was calculated according to the results obtained by Cell-ELISA on monolayers infected with CHIKV/Col. **D.** The replication percentage was calculated according to the results obtained by RT-qPCR of monolayers infected with ZIKV/Col. **E.** Viral protein percentage was calculated according to the results obtained by cell ELISA on monolayers infected with ZIKV/Col. The asterisks indicate statistically significant differences with respect to the control without compound (*p < 0.05, **p < 0.01 and ***p < 0.001; Student’s *t*-test), and error bars indicate the standard error of the mean; n: 4

The number of genomic copies/ml and viral protein production were quantified in Vero, U937 or A549 monolayers that were infected and after, treated with compounds that inhibited the production of infectious viral particles in the post-treatment strategy in each viral model (Fig. 6A). As previously shown, rupicoline had antiviral activity in the CHIKV/Col model in Vero and A549 cells; then, the number of genomic copies/ml was measured in these cell line monolayers. In Vero cells, post-treatment increased the number of genome copies/ml by 513.5% compared with the untreated control, while in A549 cells, there were no significant differences with respect to the untreated control (Fig. 6B). Meanwhile, despite increased genomic copies/ml in Vero cells with rupicoline post-treatment, viral protein production in this cell line was not affected since there were no significant differences with respect to the control; but in A549 post-treated monolayers, rupicoline decreased the production of CHIKV-viral protein to 72.6% (Fig. 6C; Table 2).

In the ZIKV/Col infection model, VOAC and VOAC-OH did not inhibit the replication of the viral genome in Vero cells; in contrast, both compounds significantly inhibited the viral genome in the U937 (3.1 and 3.0% infection rates, respectively) and A549 post-treated cells (1.6% infection rates in both cases) (Fig. 6D). Furthermore, post-treatment in ZIKV-infected monolayers with VOAC and VOAC-OH significantly reduced viral protein production in all three cell lines, Vero, U937 and A549 (Vero 0.0 and 0.0%, U937 0.0 and 0.0%; and A549 1.2 and 7.1%, respectively) (Fig. 6E; Table 2).

In all cases, ribavirin was the inhibition control. In the CHIKV/Col model, ribavirin in Vero and A549 cells reduced the infection rate to 31.3 and 53.2% and protein production to 24.0 and 30.8%, respectively. In the ZIKV/Col model, ribavirin in Vero, U937 and A549 cells reduced the infection rate to 8.6, 43.1 and 15.7% and protein production to 3.2, 3.2 and 10%, respectively.

### VOAC, VOAC-OH and rupicoline inhibited the production of infectious viral particles in the combined strategy for both viral models

**Fig. 7:**
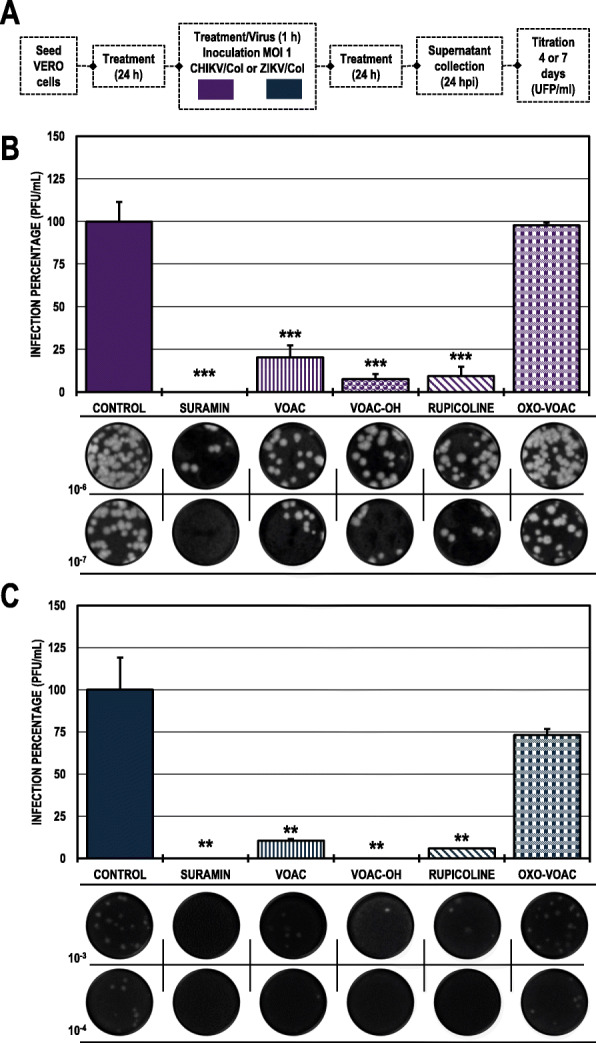
Antiviral activity on the production of infectious viral particles from cultures of Vero cells treated with the combined strategy**. A.** Schematic representation of the combined-treatment strategy explained in the Materials and Methods section. **B.** Infection percentage calculated according to the results obtained by plaque assay (PFU/ml) of the supernatants collected from Vero cells infected with CHIKV/Col under each experimental condition. **C.** Infection percentage calculated according to the results obtained by plaque assay (PFU/ml) of the supernatants collected from Vero cells infected with ZIKV/Col under each experimental condition. The asterisks indicate statistically significant differences with respect to the control without compound (*p < 0.05, **p < 0.01 and ***p < 0.001; Student’s *t*-test), and error bars indicate the standard error of the mean; n: 4. Representative images of plaques formed under each experimental condition are shown (**B** and **C**)

In the combined strategy (treatment before, during and after the infection) (Fig. 7A), three of the four indole alkaloids inhibited the production of infectious viral particles in both viral models, VOAC, VOAC-OH and rupicoline. In the CHIKV/Col model, the infection percentages were less than 25% in all cases and 20.3, 7.6, and 9.5%, respectively (Fig. 7B). Meanwhile, in the ZIKV/Col model, infection rates were less than 11% (10.5, 0.0, and 5.9%, respectively) (Fig. 7C). In Vero cells, VOAC and VOAC-OH were the compounds in the CHIKV/Col model that improved its antiviral activity with the combined strategy when compared to the individual strategies, while in ZIKV, the three alkaloids in this cell line improved their activity with the combined strategy. Suramin was the inhibitory control in both infection models, with inhibition rates of 0.0% in both cases (Table 2).

### Indole alkaloids favorably interact with one structural and nonstructural viral protein of CHIKV and ZIKV

**Fig. 8:**
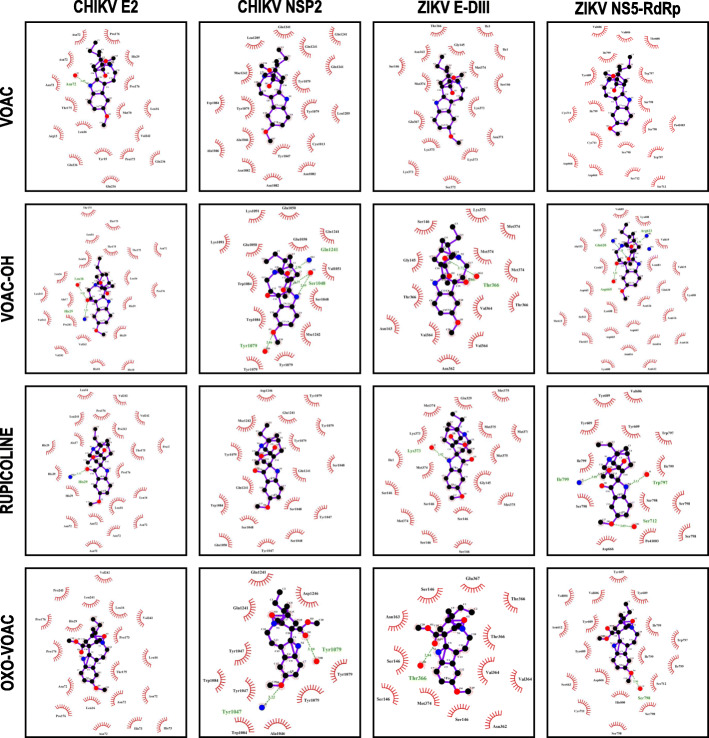
Molecular docking between each compound and structural (E2 from CHIKV and E-DIII from ZIKV) and nonstructural proteins (NSP2 from CHIKV and NS5-RdRp from ZIKV). The interactions formed were evaluated by LigPlot®. Hydrogen bonds are shown by dashed lines in green and hydrophobic interactions in red eyelashes

To evaluate the possible activity of the studied compounds on viral proteins, in silico molecular docking was evaluated for each compound with a structural viral protein and a nonstructural protein of CHIKV and ZIKV. In the CHIKV models, all the compounds had favorable binding energies with the structural protein E2 (− 6.50 ± 0.17 Kcal/mol to − 7.37 ± 0.06 Kcal/mol) and with the nonstructural protein NSP2 (− 6.00 ± 0.10 Kcal/mol to − 8.07 ± 0.06 Kcal mol); likewise, for ZIKV protein E domain III (− 5.30 ± 0.10 Kcal/mol to − 6.23 ± 0.06 Kcal/mol), and ZIKV NS5 RNA-dependent RNA polymerase (RdRp) domain (− 6.43 ± 0.06 to − 7.17 ± 0.06 Kcal/mol for protein domain). OXO-VOAC displayed the best binding energies among all evaluated proteins (Table 3). For rupicoline, which had the best in vitro antiviral activity in post-treatment strategy in the CHIKV model, a binding energy of − 6.00 ± 0.10 Kcal/mol was obtained with the NSP2 protein, without hydrogen bonds but with 15 hydrophobic interactions with eight amino acids (Fig. 8). Otherwise, when performing the molecular coupling between Domain III of the ZIKV envelope protein and VOAC-OH, the only compound with virucidal activity, the highest binding energies with this protein were obtained (− 5.57 ± 0.06 Kcal/mol). Meanwhile, VOAC and VOAC-OH, the compounds with the best in vitro activity in the post-treatment strategy against ZIKV, had binding energies of − 6.43 ± 0.06 Kcal/mol and − 7.07 ± 0.06 Kcal/mol for the nonstructural protein NS5 RdRp domain. Of these two, only VOAC-OH favorably formed 3 hydrogen bonds with amino acids Arg623 and Gln620, located in the hydrophobic pocket of the NS5 protein, and with Asp665, which is part of the catalytic triad (GDD) (Fig. 8, Table 3).

**Table 3:** Docking scores and in silico interactions predicted for interactions between indole alkaloids and viral proteins from CHIKV and ZIKV

Ligand	Virus	Protein	Free binding energy (Kcal/mol)	Hydrogen bonds	Residues forming H bonds	Distance between H+ bonds (Å)	Residues participating in hydrophobic interactions
*VOAC*	*CHIKV*	*E2*	-6,70 ± 0,10	1	Asn72	2,85	Asn72(×3)-Pro176(×2)-His29-Thr175-Met70-Leu16 (× 2)-Arg13-Val242-Gln236 (× 3)-Tyr15-Pro173
		*NSP2*	−7,03 ± 0,06	0	N/A	N/A	Gln1241 (×4)-Leu1205 (× 2)-Tyr1079 (× 3)-Cys1013-Tyr1047-Trp1084-Ala1046 (× 29-Asn1082 (× 3)
	*ZIKV*	*E DIII*	−5,30 ± 0,10	0	N/A	N/A	Thr366-Ile1 (× 2)-Asn163-Gly145-Ser146 (× 2)-Met374 (× 2)-Lys373(× 4)-Glu367-Ser372-Asn371
		*NS5*	−6,43 ± 0,06	0	N/A	N/A	Val606-Thr608-Ile799-Tyr609-Trp797 (× 2)-Ser798 (× 3)-Cys711 (× 2)-Asp666(× 2)-Ser712 (× 2)
*VOAC-OH*	*CHIKV*	*E2*	− 6,87 ± 0,06	2	Leu16-His29	3,18-3,21	Thr175 (× 4)-Leu16 (× 4)-Asn72-Pro176-Leu241-Ala17-His29 (× 2)-His18 (× 2)-Val242 (× 3)-Pro243
		*NSP2*	−6,63 ± 0,06	4	Tyr1079-Ser1048(× 2)-Gln1241	2,86-(2,89-3,25)-2,96	Glu1050 (×3)-Lys1091 (× 2)-Gln1241-Val1051-Trp1084 (× 2)-Ser1048-Tyr1079 (× 2)
	*ZIKV*	*E DIII*	−5,57 ± 0,06	1	Thr366	2,71	Gly145-Ser146-Thr366 (× 2)-Lys373-Met 374(× 3)-Val364 (× 3)-Asn163-Asn362
		*NS5*	−7,07 ± 0,06	3	Arg623-Gln620-Asp665	2,91-3,28-3,12	Val689-Lys688-Val619 (× 2)-Ala533-Leu683-Cys667-Asp665 (× 3)-Gln620-Asn616 (× 5)-Lys688-Ile543-Thr615 (× 2)-Asn612
*RUPICOLINE*	*CHIKV*	*E2*	−6,50 ± 0,17	1	His29	3,11	Leu16 (×3)-Val242(×2)-Pro176 (× 2)-Pro243-Pro173-Thr175-Leu241-His29 (× 3)-Ala17-Asn72(× 5)
		*NSP2*	−6,00 ± 0,10	N/A	N/A	N/A	Asp1246-Tyr1079 (× 3)-Gln1241 (× 3)-Tyr1079-Ser1048 (× 4)-Tyr1047 (× 2)-Trp1084-Glu1050
	*ZIKV*	*E DIII*	−5,40 ± 0,10	1	Lys373	2,97	Met375 (×5)-Glu 329-Met374 (× 3)-Lys373-Ile1-Ser146 (× 5)-Gly145.
		*NS5*	−6,57 ± 0,21	3	Ile797-Ser712-Trp797	3,09-3,05-3,13	Val606-Tyr609 (× 3)-Trp797-Ile799 (× 2)-Ser798 (× 4)-Asp666
*OXO-VOAC*	*CHIKV*	*E2*	−7,37 ± 0,06	N/A	N/A	N/A	Val242 (×2)-Pro243-Leu241-Leu16 (× 3)-Pro176 (× 3)-Pro173-His29-Thr175-Asn72 (× 4)-His73 (× 2)
		*NSP2*	−8,07 ± 0,06	2	Tyr1079-Tyr1047	3,10-3,22	Gln1241 (× 2)-Tyr1047 (× 2)-Asp1246-Tyr1079 (× 2)-Ala1046-Trp1084 (× 2)
	*ZIKV*	*E DIII*	−6,23 ± 0,06	1	Thr366	2,84	Glu367-Ser146 (×4)-Asn163-Thr366 (× 2)-Val364 (× 2)-Asn362-Met374
		*NS5*	−7,17 ± 0,06	1	Ser798	2,96	Tyr609 (× 4)-Val606 (× 2)-Ile799 (× 3)-Trp797-Ser712-Asn612-Ser663-Asp666-His800-Cys711-Ser798-Ser798

### The interaction between the E protein of ZIKV and VOAC-OH is partially stable over time via molecular dynamics

**Fig. 9:**
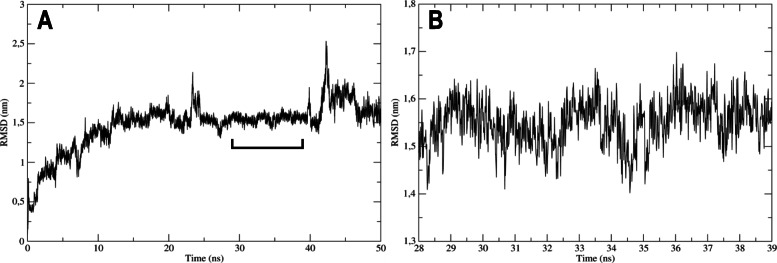
Complex stability by molecular dynamics. **A.** The stability of the complex formed by the compound with virucidal effect (VOAC-OH) and Domain III of ZIKV envelope protein was evaluated during 50 ns of simulation. **B.** The site with the lowest oscillation of simulation is shown. The y-axis represents the root mean square deviation (RMSD), which represents the average oscillation of the distance between the atoms of the complex components in nanometers (nm), and the x-axis represents the timescale in nanoseconds (ns). The complex is considered stable if the oscillation from the initial position of the complex is below 0.3 nm

The analysis of molecular dynamics included the oscillating distance between the ligand and the target protein during the time assessed (50 ns). For the complex to be considered biologically feasible, the distance must be less than 3 Å or 0.3 nm. The RMSD of the simulation between domain III of the ZIKV envelope protein and VOAC-OH was between 0.5 nm and 2.5 nm, indicating an oscillation of 2 nm (20 Å). Then, the complex was not stable over 50 ns (Fig. 9A). Despite this, the RMSD between 28 and 39 ns had an oscillation < 0.3 nm and could be considered stable at this time (Fig. 9B).

## Discussion

Considering that there is still no specific antiviral treatment for infections caused by CHIKV and ZIKV and that these viruses continue to co-circulate around the world, research focused on the search for these types of compounds through different experimental approaches remains a priority. In this study, we evaluated the in vitro and in silico antiviral activity of four indole alkaloids against these two arboviruses.

As indole alkaloids were not cytotoxic, it was possible to proceed with their antiviral evaluation (Table 1). The first evaluated antiviral strategy (pre-treatment) showed significant inhibition only with VOAC and VOAC-OH in the CHIKV/Col infection model (Fig. 1B). Although the structural differences between the four compounds studied were small, these differences could be sufficient to generate the changes in antiviral activity observed. As with these tryptophan-derived compounds, other amino acid-derived antivirals, such as dihalogenated antivirals derived from L-tyrosine, have been described that those small differences in molecules can generate changes in activity and affinity for their therapeutic mechanism [28]. Likewise, curcumin and its analogs bisdemethoxycurcumin and desmethoxycurcumin, which can increase or decrease the anti-CHIKV activity with small structural changes using the same experimental strategy [36]. Nonetheless, it has been previously reported that VOAC in pre-treatment strategy did not inhibit the production of viral particles in the CHIKV infection model [26], a result that contradicts the findings reported in the present study. However, this difference could be explained by the fact that in the previous study, the infection used a different MOI, and it has been reported that compounds such as chloroquine, with known activity in the early stages of infection, may have MOI-dependent activity in the influenza A virus A/WSN/1933(H1N1) [37]. Additionally, the alkaloid tomatidine has shown a MOI-dependent effect in another arboviral infection model, ZIKV, where inhibition at a higher MOI (10) had an antiviral effect but did not have significant inhibition at a lower MOI (5) [38].

In contrast, in the ZIKV infection model, three of the four alkaloids (VOAC, VOAC-OH, and OXO-VOAC) had an antiviral effect using this same strategy (Fig. 2B). The activity of these three compounds and not of rupicoline could be associated with the structural differences, that have also been reported in curcumin, bisdemethoxycurcumin, and desmethoxycurcumin in the ZIKV model [36]. Moreover, although the inhibition in early stages has been associated with larger molecules, small alkaloid molecules with the ability to inhibit early infection processes have been also described; for example, the adhesion of the particle to the CCR5 cell receptor in the HIV model with maraviroc [39] and BMS663068 [40] or the inhibition of endosome acidification in the CHIKV model with compounds such as niclosamide and chloroquine [41]. Such mechanisms of action could be proposed for the alkaloids studied here, which would indicate that the compounds act as cellular receptor antagonists and block interaction with the envelope proteins of both models or modify cellular processes involved in the viral replicative cycle.

Next, the compounds that showed antiviral activity in Vero cells in other biologically relevant human cell models (monocytes and fibroblasts) were evaluated using the same antiviral strategy (Fig. 1A). According to the above, the results revealed that only one compound, VOAC-OH, inhibited infection in U937 cells infected with CHIKV/Col, whereas none of the compounds had an effect on A549 cells (Fig. 1C). This result could be supported by the fact that different types of cells respond in their own way depending on the viral infection [42, 43]. Thus, the compounds in each cell type could have different metabolisms, resulting in a cell antiviral response that is dependent on the cell line. Contrary to what was observed in CHIKV/Col, in the ZIKV/Col model, the three compounds effective in Vero cells, VOAC, VOAC-OH and OXO-VOAC, were also effective in U937 and A549 cells (Fig. 2C), demonstrating that the activity of these three indole alkaloids is independent of the cell line in this in vitro ZIKV/Col infection model. The differences between the two viral models could be explained by the differences in the entry receptors used by both viruses [44, 45] and by the fact that infection by each arbovirus model modulates the signaling pathways of the host cell differently [42].

On the other hand, virucidal activity was found only with treatment with VOAC-OH against ZIKV/Col (Fig. 3). It has been previously described those natural compounds with a structural relationship may exhibit differences in their virucidal capacity, as is the case for 4′-O-methylepigallocatechin and proanthocyanidin against the Mayaro virus. The first compound does not present activity, even at the highest concentrations, whereas the second presents potent virucidal activity against this arbovirus [46]. Likewise, it has been reported that epigallocatechin gallate, which is structurally related to the two previously mentioned compounds, has virucidal activity, acting directly on the ZIKV envelope [47]. Furthermore, it has been reported that a given compound can have opposite effects in two different viral models; such is the case for the alkaloid harringtonine derived from *C. harringtonia*, which has virucidal activity against ZIKV [48] but not against CHIKV [49]. Moreover, another structurally related alkaloid derived from the same plant, cephalotaxine, also appears to have virucidal activity in the same model (ZIKV) [50].

These results may be related to what has been obtained in studies showing that related compounds may have different activity within the same viral model, and related compounds may also have activity on a different viral model.

For the post-treatment strategy in Vero cells infected with CHIKV/Col, only the indole alkaloid rupicoline inhibited the production of infectious viral particles (Fig. 4B). This inhibitory effect was observed in A549 cells but not in U937 cells (Fig. 4C). Again, this is related to what is described above, where small structural changes in a molecule can lead to the creation or loss of the antiviral activity of a compound [51]. This could be related to the structural difference of rupicoline compared with the other three alkaloids in the study, as rupicoline presents a break in the heptagonal ring structure [27]. This difference appears to be the determining factor in the anti-CHIKV activity observed in the present study. Joined to this, the cell dependence of rupicoline could be related to the differences presented in the signaling and metabolic processes of each type of cell line infected with CHIKV [42, 43, 52, 53]. This behavior of the cell line-dependent anti-CHIKV response has been described on small molecule compounds such as ribavirin and favipiravir. At concentrations below 100 μg/ml, ribavirin antiviral activity was observed in HUH-7 cells but not in Vero or A549 cells, and at concentrations below 19.64 μg/ml, favipiravir did not display any anti-CHIKV activity in A549 cells but displayed this activity in Vero and HUH-7 cells [54]. In contrast, when evaluating the viral genome and protein production using this same strategy, in the Vero cells infected with CHIKV/Col and subsequently treated with rupicoline, there was an accumulation of viral RNA that did not affect the production of viral protein, whereas in the A549 cells, there were no changes in the viral RNA and there was inhibition of the protein production (Fig. 6B-C). Then, we hypothesize that the activity of rupicoline is directed not only to viral proteins but also to cellular processes related to viral replication. Previous studies on other compounds, such as statins, have shown that a possible mechanism of antiviral action occurs during the late stages of infection, such as the assembly, maturation, or release of the viral particle [55]; therefore, according to the results obtained with rupicoline, it is suggested that its effect in Vero cells is found in steps subsequent to the replication of the viral genome and translation of the viral proteins involving cellular processes specific to each line.

In the ZIKV model, the inhibition of infectious viral particles and protein production in cultures post-treated with VOAC and VOAC-OH was independent of the cell lines studied (Fig. 5B-C and Fig. 6E), but only in Vero cells was there no inhibition of the viral genome (Fig. 6D). This same result has been described for the alkaloid cephalotaxine, for which a post-treatment with concentrations of 20 μM ZIKV-infected cultures showed no inhibition of ZIKV RNA in Vero cells despite decreasing the viral load (FFU) [50]. Similarly, post-treatment with harringtonine at a concentration of 156 μM resulted in inhibition of ZIKV (FFU), viral load, and viral protein but not of RNA in Vero cells [48]. Additionally, it has been shown that indole alkaloids such as arbidol and homoharringtonine are capable of activating the type I IFN response [56, 57] and that Vero is a cell line that lacks IFN-alpha genes, a fundamental component in the innate antiviral response. IFN is induced in response to the presence and identification of viral or double-stranded RNA within the cell and subsequently activates INF-stimulated genes, such as PKR, 2′-5′ oligoadenylate synthetases, and RNase L, among others [58], which could degrade viral RNA [59]. Taking the above into account, this could be the reason for the difference found in the inhibition of the genome between Vero cells and human cell lines U937 and A549. Future studies of modulation of the INF response by these compounds could clarify this hypothesis.

The last in vitro antiviral evaluation was the combined strategy, which showed that among the four indole alkaloids evaluated, only VOAC, VOAC-OH and rupicoline significantly inhibited the output of infectious viral particles in the two viral models (Fig. 7). The ability of a compound to inhibit the infection of an alphavirus and a flavivirus has been previously described, as is the case of atovaquone [60], quinolone-N-acylhydrazone [61], coumarins A and B, also derived from *T. cymosa* [26] and dihalogenated derivatives of L-tyrosine [28]. Evidence of activity in both viral models with multiple factors in common, such as symptoms in the acute phase, transmission by the same vector, and co-circulation in the same areas [2, 62, 63], makes this result biologically relevant and encouraging in the search for broad-spectrum antivirals for these pathologies. Although the combined antiviral strategy indicates whether the compound has an inhibitory effect, it does not contribute to the explanation of a possible antiviral mechanism; therefore, despite this model being clinically closer, evaluation of the previously described strategies is required. Alkaloids have been described as having multiple antiviral mechanisms of action. Such is the case for the alkaloid lycorine, which can inhibit replication of the flavivirus hepatitis C virus by inhibiting proteins such as HSP70, ZIKV RdRp and 2A protease of EV71A [64]. Therefore, in some cases, the activity in the combined strategy, such as rupicoline in the ZIKV/Col model, could mean the synergy of direct mechanisms of action against viral proteins and of cellular proteins involved in viral replication in the continuous presence of the compound, contrary to the findings in other cases in which the combined strategy is similar to that of the individual strategies.

Finally, the use of biocomputational tools, such as molecular docking, has become increasingly important not only for the discovery of drugs [65] but also to model the interaction between a molecule and a protein at the atomic level. This allows the characterization of the behavior of the compounds in reference to the position and orientation of the ligand in the interaction pocket and the evaluation of the binding affinity [66], which helps explain the possible mechanisms of action. To contrast the in vitro results and to reinforce the already discussed mechanisms of action, we evaluated the in silico activity of the indole alkaloids studied here on a structural protein (envelope) and a nonstructural protein from each of the viruses (NS5 polymerase domain and NSP2 protease domain, for ZIKV and CHIKV, respectively). Favorable energies were obtained for rupicoline, the only alkaloid with post-treatment activity in the CHIKV model, but the mechanism of action could not be related to the viral protein because neither the genomic copies nor the viral protein in Vero cells were inhibited. Cellular processes could therefore be involved in this compound’s mechanism of action. VOAC and VOAC-OH were the compounds with the best in vitro antiviral activity in ZIKV; they interacted favorably with the polymerase domain of NS5, forming multiple hydrophobic interactions and even hydrogen bonds in the case of VOAC-OH (Fig. 8), which could indicate direct activity against this protein that could correlate with the decrease of the viral genome, as seen in the U937 and A549 cells (Fig. 6B and D), and that could, in turn, lead to the decrease of the viral protein observed in all cell lines (Fig. 6C and E). The importance of envelope glycoprotein has been described for the entry of the virus into the cell [67]. In this study, only the result obtained with VOAC-OH in ZIKV could be related to their in vitro activity, given that they had favorable binding energy with the structural protein and virucidal activity also, which could be related to the possible mechanism of action of this compound. To confirm this result, a fluid simulation of the interaction complex formed by domain III of the ZIKV envelope (E-DIII) and VOAC-OH by molecular dynamics was performed. The results indicate that there was partial stability, with no stability over 50 ns, but stability for more than 10 ns in part of the simulation (Fig. 9), suggesting that the possible action mechanism could be related but does not focus solely on the interaction between protein E-DIII and VOAC-OH (Fig. 8). Further in silico studies could elucidate whether it is an interaction between another domain of protein E or other conformations.

The methodologies used in this research work demonstrated the antiviral effect and could bring us closer to the possible mechanisms of action of the four indole alkaloids derived from *T. cymosa* evaluated. However, future studies focused on cellular biology on viral infection could help to understand the specific mechanisms involved in the differential antiviral effect.

## Conclusion

We demonstrated that indole alkaloids derived from *T. cymosa* are promising antiviral drugs in the process of ZIKV/Col and CHIKV/Col in vitro infection. Moreover, the mechanisms of action evaluated indicate that the antiviral effect is different in each viral model and, in turn, dependent on the cell line.

## Supplementary Information

**Additional file 1** Chemical structure of indole alkaloids used in the study

## Data Availability

The datasets used and analyzed during the current study are available from the corresponding author on reasonable request.

## Abbreviations

ZIKV: Zika virus
CHIKV: Chikungunya virus
VOAC: Voacangine
VOAC-OH: Voacangine-7-hydroxyindolenine
OXO-VOAC: 3-oxo voacangine
ORF: Open reading frames
C: Capside protein
prM: Membrane precursor protein
E: Envelope protein
NS1: Non-structural protein 1
NS2a: Non-structural protein 2a
NS2b: Non-structural protein b
NS3: Non-structural protein 3
NS4a: Non-structural protein 4a
NS4b: Non-structural protein 4b
NS5: Non-structural protein 5
L-15: Leibovitz medium
DMEM: Dulbecco’s modified Eagle Medium
FBS: Fetal bovine serum
PCR: Conventional polymerase chain reaction
qPCR: Quantitative polymerase chain reaction
MTT: 3-[4,5-dimethylthiazol-2-yl]-2,5 diphenyl tetrazolium bromide
Cell-ELISA: Cell-Enzyme-Linked Immunosorbent Assay
PDB: Protein Data Bank
PMV: Python Molecular Viewer
RdRp: RNA dependent RNA polymerase
MOI: Multiplicity of infection
FFU: Fluorescent focus units
PFU: Plaque-forming units
