# Supplementary figures and images for: Indole alkaloids inhibit zika and chikungunya virus infection in different cell lines
Source: BMC Complement Med Ther. 2021 Aug 28;21:216. doi: 10.1186/s12906-021-03386-z (PMC8397866; doi:10.1186/s12906-021-03386-z)

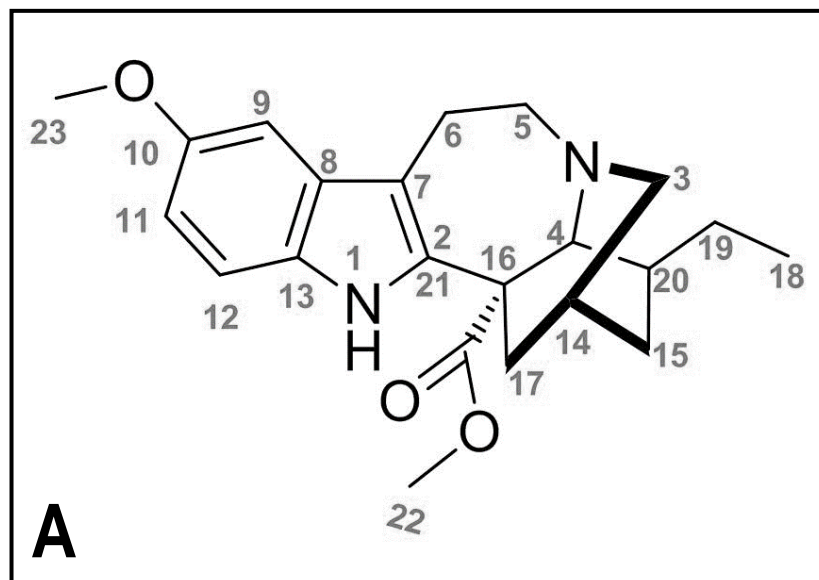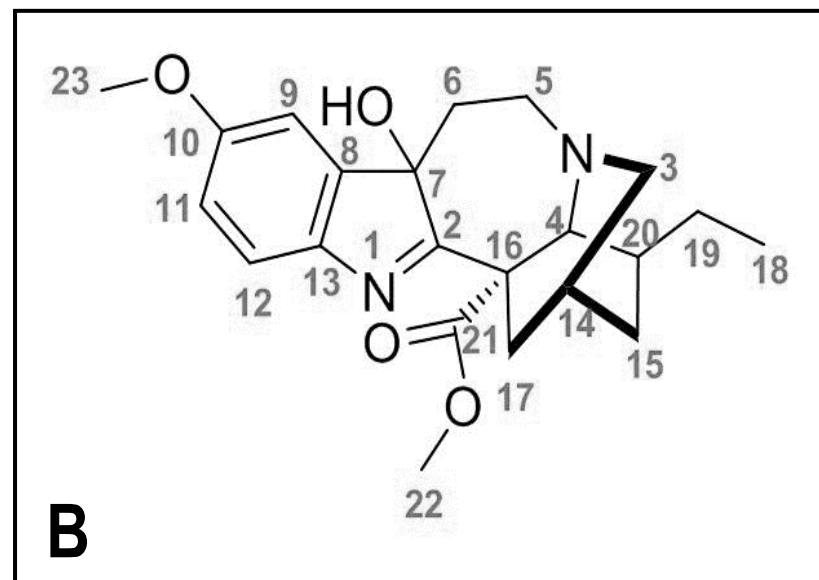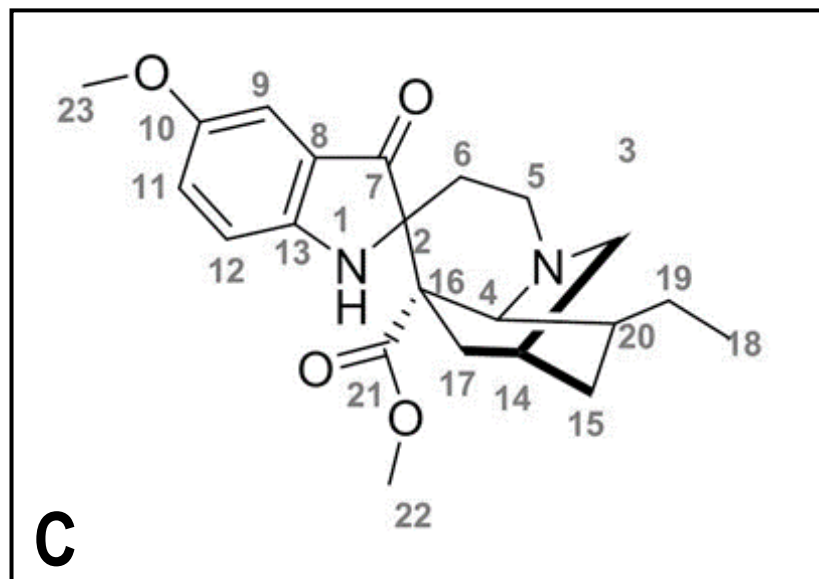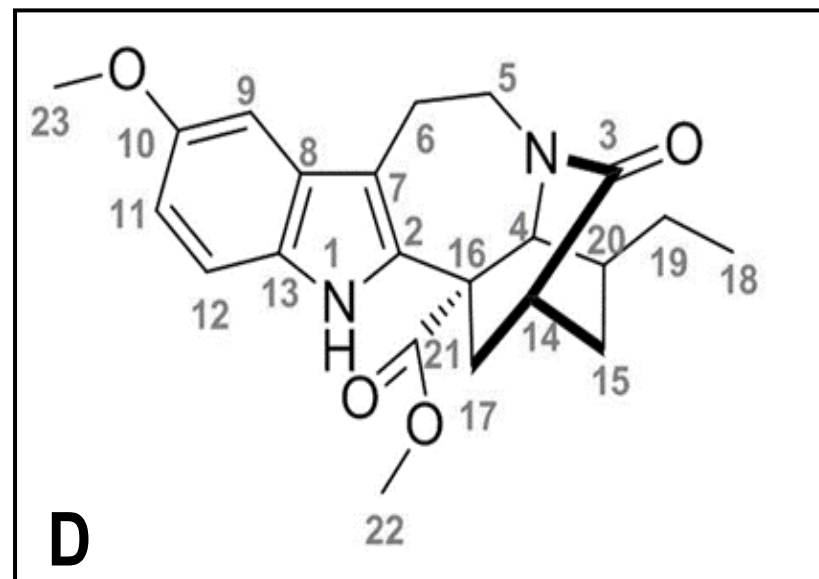

Supplement: Supplementary file 1 — Additional file 1 Chemical structure of indole alkaloids used in the study [file 12906_2021_3386_MOESM1_ESM.pdf]
